# No association between osteoporosis and AO classification of distal radius fractures: an observational study of 289 patients

**DOI:** 10.1186/s12891-020-03842-w

**Published:** 2020-12-04

**Authors:** Anja M. Hjelle, Jan-Erik Gjertsen, Ellen M. Apalset, Roy M. Nilsen, Anja Lober, Grethe S. Tell, Pawel Mielnik

**Affiliations:** 1 Department of Rheumatology, Division of Medicine, District General Hospital of Førde, Førde, Norway; 2 Department of Radiology, District General Hospital of Førde, PO Box 1000, 6807 Førde, Norway; 3 Department of Global Public Health and Primary Care, University of Bergen, Bergen, Norway; 4 Department of Orthopedic Surgery, Haukeland University Hospital, Bergen, Norway; 5 Department of Clinical Medicine, University of Bergen, Bergen, Norway; 6 Bergen group of Epidemiology and Biomarkers in Rheumatic Disease (BeABird), Department of Rheumatology, Haukeland University Hospital, Bergen, Norway; 7 Faculty of Health and Social Sciences, Western Norway University of Applied Sciences, Bergen, Norway

**Keywords:** Osteoporosis, Dual energy x-ray absorptiometry, Distal radius fracture, AO classification

## Abstract

**Background:**

It is mechanically plausible that osteoporosis leads to more severe peripheral fractures, but studies investigating associations between BMD and radiographically verified complexity of distal radius fractures are scarce. This study aims to study the association between osteoporosis, as well as other risk factors for fracture, and the AO classification of distal radius fractures.

**Methods:**

In this observational study, 289 consecutive patients aged ≥40 years with a distal radius fracture were included. Bone mineral density (BMD) of the hips and spine was measured by dual-energy x-ray absorptiometry (DXA), and comorbidities, medication, physical activity, smoking habits, body mass index (BMI), and history of previous fracture were registered. The distal radius fractures were classified according to the Müller AO system (AO) (type B and C regarded as most complex).

**Results:**

Patients with osteoporosis (*n* = 130) did not have increased odds of a more complex distal radius fracture (type B + C, *n* = 192)) (n = vs type A (*n* = 92) (OR 1.1 [95% CI 0.5 to 2.3]) compared to those with osteopenia /normal BMD (*n* = 159). Patients with AO fracture types A or C had a higher prevalence of osteoporosis than patients with type B fracture.

**Conclusions:**

Distal radius fracture patients with osteoporosis did not sustain more complex fractures than those with osteopenia/normal BMD according to the AO classification system. The AO classification of distal radius fracture cannot be used to decide which patients should be referred to DXA scan and considered for secondary fracture prevention.

## Background

Distal radius fractures are the most common of all fractures during a lifespan. A Norwegian study found an overall annual incidence of 19.7 per 10,000 inhabitants 16 years or older [1]. In women, the incidence of distal radius fractures increases progressively with age from the perimenopausal period, while in men, the incidence remains low until later in life [2, 3]. According to the Swedish fracture registry (www.frakturregistret.se), 19, 357 women over the age of 60 suffered a distal radius fracture in 2018. Distal radius fractures are closely related to low bone mineral density (BMD) [4], and risk factors for fracture also include increasing age, female sex, low body mass index (BMI), smoking, postmenopausal status, low intake of dairy products, vitamin D deficiency, and autoimmune comorbidities. Patients sustaining a distal radius fracture have an increased risk of a major osteoporotic fracture (MOF) of the hip and vertebrae later in life [5, 6]. According to guidelines of fracture liaison services, a low energy fracture in an at-risk patient (e.g. > 50 years old) should lead to further examination with dual-energy x-ray absorptiometry (DXA) and treatment with anti-osteoporotic drugs if indicated [7].

When it comes to distal radius fractures and radiographic severity a few studies have been performed [8–12], but the number of patients examined are limited, the methods used differ, and conclusions are not easily drawn. Therefore, our aim was to further investigate if there is an association between osteoporosis and other well-known risk factors for osteoporotic fractures and AO classification of distal radius fractures.

## Methods

### Subjects

From March 1, 2012 until January 13, 2017, patients aged ≥40 years presenting with acute distal radius fracture at the Department of Orthopedic Surgery at District General Hospital of Førde (Sogn og Fjordane County) were included in a case control study. The study was primarily designed to explore the prevalence of celiac disease in patients with peripheral fractures compared to community-based controls. The original study has previously been described [13]. Fracture patients who fulfilled the inclusion criteria and consented to participate were referred for DXA scan and consideration of secondary fracture prevention (*n* = 516). Two hundred eighty-nine patients agreed to participate, giving an inclusion rate of 56%. We included both patients with low energy fractures (equivalent to fall from standing height or lower) and fractures due to traumas with higher energy. Five patients suffered multiple simultaneous fractures (one with bilateral radius fractures, one with an additional humerus fracture, one with an additional ankle fracture, and two with additional vertebral compression fractures).

### Procedures and measurements

The radiographic distal radius series comprised standard anteroposterior and lateral radiographs. In 68% of cases (197 of 289 patients) a supplementary CT scan was available. The same radiologist classified the fractures as extra articular (type A), partly articular (type B) or complete articular (type C) according to the Müller AO-system (AO) [14, 15]. Types B and C were considered more complex than type A. In addition, the multifragmentary fractures (types A3, C2 and C3) combined were compared to the other AO fracture types. Five of the distal radius fractures could not be classified because the radiographic images had been performed elsewhere.

The BMD measurements were performed by DXA technology (Lunar Prodigy Rtg 5603, manufacture year 2000, GE Healthcare), with a daily quality assurance of +/− 2%. BMD was reported as g/cm^2^ and T-scores by standard definition. Osteoporosis is defined as T-score ≤ − 2.5 in the femoral neck, total hip or lumbar spine. Osteopenia (low bone mineral density) is defined as T-score between − 1.0 and − 2.5 [16]. History of previous fractures, comorbidities, medications, and lifestyle factors were registered. The original documents from the orthopedic surgeons and examining rheumatologist were reviewed to classify the injury as due to a low energy trauma or not. Height and weight were measured as part of the DXA procedure. BMI was calculated and categorized into underweight (BMI < 18.5), normal weight (BMI 18.5–24.99), overweight (BMI 25–29.99) and obesity (BMI ≥ 30). Blood tests were analyzed to detect common causes of secondary osteoporosis [13].

### Statistical analyses

We performed descriptive statistics for age, sex, BMI, number of patients with osteoporosis, osteopenia, and overweight in the distal radius fracture subgroups. Data between subgroups were compared using chi square or Fisher’s exact test for categorical data and two-sample t-test or Mann-Whitney U test for continuous data. To assess risk factors associated with the complexity of fractures, we estimated odds ratios (ORs) with 95% confidence intervals (CIs) using unconditional logistic regression models. Complexity of fractures was defined as more radiological complex fractures (AO type B + C) as opposed to less complex fractures (AO type A). Relevant risk factors for complexity of fracture included osteoporosis, osteopenia, age > 65 years, male sex, BMI, and current and previous smoking. In all analyses, the association between the risk factor and the complexity of fractures was first examined crudely and then with adjustment for the other risk factors under study. All *p*-values were two-sided and values below 0.05 were considered statistically significant. All calculations were performed using R version 3.6.2 (team).

## Results

**Fig. 1:**
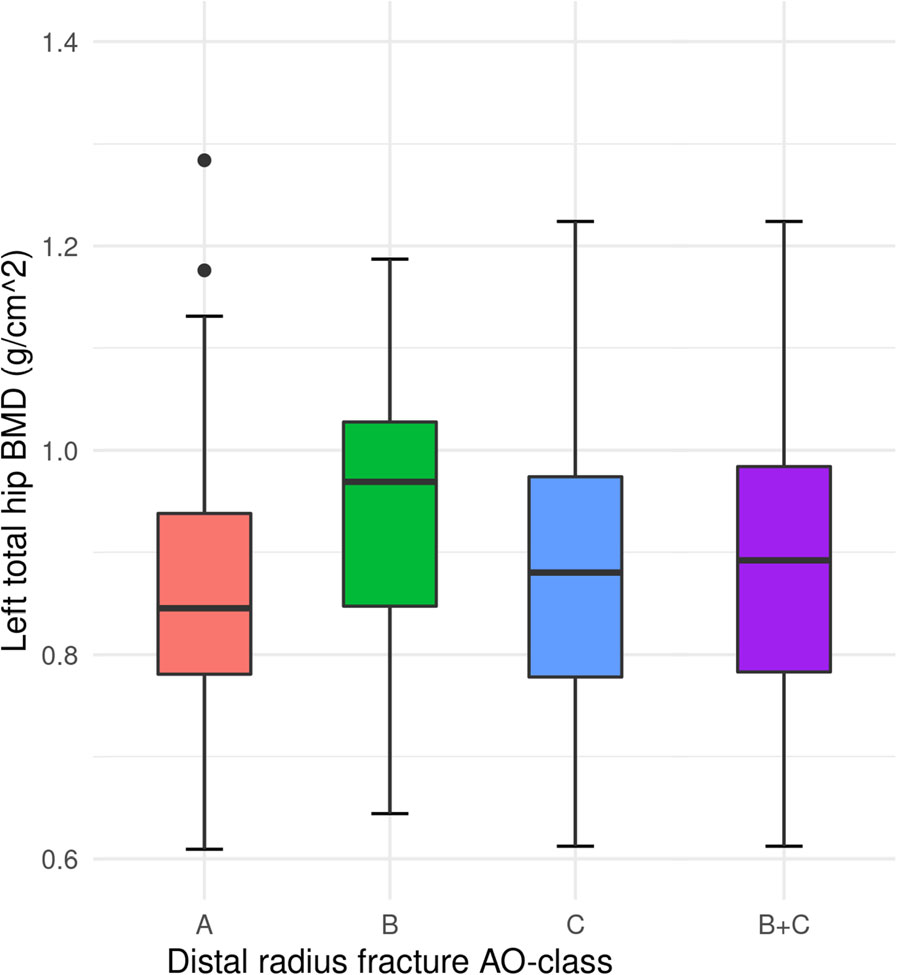
Left hip total BMD measurements box plot for distal radius fracture subgroups.AO, AO classification. Centre horizontal line of the boxes represents the median. The boxes contain Q1 (25th Percentile) to Q3 (75th Percentile). IQR (Interquartile range) is the distance between Q1 and Q3. The bottom whiskers: less than Q1–1.5*IQR. The upper whiskers: greater than Q3 + 1.5*IQR. BMD measurements in 9 patients missing (left hip not measurable)

We found that 45.0% (*n* = 130) of patients with distal radius fracture had osteoporosis and 33% (*n* = 95) had osteopenia (Table 1). Patients with an AO type B fracture were younger, had a higher mean BMI, and the percentage of men was higher than in the groups with A or C fractures (Table 1). 29.4% of patients with type B fracture had osteoporosis compared to 46.7% of type A and 48.1% of type C (Table 1, Fig. 1). The patients with osteoporosis differed from the patients with osteopenia/normal BMD at a group level by having a statistically lower BMI (BMI 26 vs 27, *p*-value 0.005), being older (mean age 64 vs 53 years, *p*-value 0.01), a greater percentage were female (88% vs 75%, p-value 0.01), and there was a higher prevalence of current smoking (18% vs 13%, p-value 0.1). There was a significantly higher proportion with low energy trauma mechanism in the patients with radius fracture and osteoporosis compared to those with radius fractures and normal BMD/osteopenia (77% vs 57%, p-value < 0.001).

**Table 1:** Characteristics of patients with distal radius fractures according to type of fracture (Müller AO classification system)

	Fracture type				
	All	AO type A	AO type B	AO type C	AO type B + C
Total n	289	92	34	158	192
Age, mean (range)	63 (40–92)	62 (42–88)	62 (42–80)	64 (40–92)	64 (40–92)
Female sex, n (%)	231 (80)	78 (85)	21 (61)	128 (81)	149 (78)
Osteoporosis^a^, n (%)	130 (45)	43 (47)	10 (29)	76 (48)	86 (45)
Osteopenia^b^, n (%)	95 (33)	31 (34)	13 (38)	47 (30)	60 (32)
BMI, mean (SD)	26 (5)	26 (4)	28 (5)	26 (5)	26 (5)
Overweight, n (%)	95 (33)	27 (29)	12 (35)	55 (35)	67 (35)
Obesity, n (%)	64 (22)	21 (23)	11 (32)	28 (18)	39 (21)
Current smoking, n (%)	43 (15)	20 (22)	3 (9)	19 (12)	21 (11)
Previous smoking, n (%)	121 (42)	39 (42)	11 (32)	67 (42)	64 (41)

^a^ T-score ≤ − 2.5

^b^ T-score − 1.0 - -2.5

*AO* AO classification, *BMI* Body Mass Index (BMI categories: underweight BMI < 18.5, normal weight BMI 18.5–24.99, overweight BMI 25–29.55 and obesity BMI ≥30.0); SD: Standard deviation

The OR of sustaining a distal radius fracture type B or C vs. A was not significantly affected by the presence of osteoporosis (Table 2). Current smoking and low energy trauma injury were associated with less complex fractures (Table 2). When combining the multifragmentary fractures across the classification groups (A3 + C2 + C3), the OR of sustaining a multifragmentary fracture did not significantly differ according to BMD status (osteoporosis gave an OR of 1.4 (95% CI 0.6–3.7), and osteopenia OR 1.0 (95% CI 0.4 to 2.6)). Low energy trauma mechanism decreases the odds of comminuted fractures compared to the other AO subgroups (OR for (A3 + C2 + C3) 0.3 (95% CI 0.1–0.5)).

**Table 2:** Odds Ratios (unadjusted and adjusted) for complex (AO type B and C) vs. less complex (AO type A) distal radius fractures

	OR with 95% CI	
Exposures	Unadjusted	Adjusted
BMI	1.0 (0.8–1.3)	1.0 (0.7–1.3)
Current smoking^a^	0.4 (0.2–0.9)	0.4 (0.2–0.8)
Previous smoking^a^	0.8 (0.4–1.3)	0.7 (0.4–1.3)
Osteoporosis^b^ T-score ≤ −2.5	0.8 (0.4–1.5)	1.1 (0.5–2.3)
Osteopenia^c^ T-score − 1.0 - -2.5	0.8 (0.4–1.6)	1.0 (0.5–2.1)
Age > 65 years^d^	1.4 (0.9–2.4)	1.6 (0.9–2.7)
Male sex^e^	1.7 (0.9–3.5)	1.5 (0.7–3.1)
Low energy trauma^f^	0.5 (0.3–0.8)	0.4 (0.2–0.8)

*OR* Odds ratio, *CI* Confidence interval, *BMI* Body mass index

Relevant risk factors adjusted for were: age, sex, BMI, smoking, bone mineral density and low energy trauma

^a^Reference category was the non-smoking group. When analyzing current smoking, the group of previous smoking is removed, and vice versa

^b^Reference category was no osteoporosis (osteopenia and normal bone mineral density)

^c^Reference category was normal bone mineral density (T-score ≥ − 1.0)

^d^Reference category was age < 65

^e^Reference category was female sex

fReference category was no low energy trauma

## Discussion

The odds of sustaining a distal radius fracture Type B or C compared to Type A in patients with osteoporosis did not differ from those with osteopenia or normal BMD. This indicates that the AO classification of the fracture cannot be used to decide which patients should be referred to DXA scan and considered for secondary fracture prevention. One may argue that the AO-classification system is not able to capture all the facets of a fracture, as many factors concern the mechanical complexity and etiology of a fracture (e.g. the position and angle of the extremity and the body at the time of the fall, body composition and weight, balance, rotational forces, and the surroundings). A more detailed discussion of the classification system is beyond the scope of the current study, which aims to investigate the association between osteoporosis and the severity of distal radius fractures using established radiographic methods.

Our results are in line with previous rapports. A study including 137 patients with low-energy distal radius fractures found an inverse correlation between BMD of the hip measured 3 months after the fracture and likelihood of early instability, late carpal malalignment and malunion [10]. However, no correlation between BMD and the AO subtypes was found. The same study found that BMD in patients with type C fractures was higher than in patients with type A fractures, which is in agreement with our results. This is also supported by a study of 208 patients with distal radius fracture, where no correlation between the AO-classification and BMD of the hips and spine was found [11]. The authors suggested that a possible explanation for this might be that DXA measures thickness of cortical bone, which is thicker in the metaphyseal area than in the epiphyseal area. A more severe osteoporotic fracture would therefore be a metaphysal fracture instead of an intra-articular fracture. Dhainaut et al. [12] assessed cortical hand BMD by digital X-ray radiogrammetry in 110 female patients with fragility fracture at the distal radius, and concluded with no correlation between neither BMD of the hip or spine nor the digital X-ray radiogrammetry and the AO fracture type. The only significant risk of intra-articular distal radius fracture compared to less complex fractures in that study was ever having used glucocorticoids, supporting the hypothesis that the severity is more associated with other factors comprising bone quality than BMD.

Severity of a distal radius fracture is a clinical assessment. The AO classification does not take into consideration instability, malunion, decreased radial length or the degree of dorsal angulation. It is clinically not clear if a complete articular fracture without displacement (C1) is more harmful to the patient than an extra-articular fracture with metaphyseal comminution (A3). Clayton et al. [10] define the most serious distal radius fracture types as A3, C2 and C3. Subanalysis of our data did not support that osteoporosis leads to a higher proportion of these fractures compared to other subtypes. We found a significantly lower OR for low energy trauma among those with type B or C fracture compared to type A. This illustrates that factors influencing fracture severity may be complex.

### Strengths and limitations

Our study was not primarily designed to investigate the association between osteoporosis and radiological severity of distal radius fractures. The study was therefore underpowered to conclude on some aspects, as there are many subtypes of fractures and accordingly few fractures in some of the groups. We did, however, have a large number of patients compared to previous studies, and we included both women and men. The radiographic interpretations were done by an experienced radiologist, and the AO classification has earlier been shown to have good intra-observer reliability when restricted to the three main AO-types [17]. To our knowledge no studies have shown an association between the AO-classification, fracture severity and clinical outcome. Accordingly, based on our results the clinical severity of the fractures could not be assessed, only the radiographic complexity. A strength of this study was the availability of supplementary CT scans in 68% of the distal radius fractures. The use of CT scans may explain that there were more AO type C fractures in our study, compared to other studies reporting more type A fractures.

## Conclusions

In this study, severity of distal radius fractures according to the AO-classification of distal radius fractures was not associated with osteoporosis when adjusted for age, sex, and BMI. AO-classification of distal radius fractures cannot be used to identify which patients should be evaluated and treated for osteoporosis.

## Data Availability

Due to regulations from the Norwegian Data Inspectorate and according to Norwegian personal protection laws publication of the complete dataset is not legal or appropriate. If authors/researchers wish to have access to the dataset, this can still be achieved through direct contact with us.

## Abbreviations

BMD: Bone mineral density
DXA: Dual-energy x-ray absorptiometry
BMI: Body mass index
AO: Müller AO classification system
MOF: Major osteoporotic fracture
